# Post-Esophagectomy Chylothorax with Thoracic Duct Anomaly Successfully Treated with Lymphangiography: A Case Report

**DOI:** 10.70352/scrj.cr.24-0129

**Published:** 2025-04-25

**Authors:** Akihiro Kuroda, Sho Yajima, Masayuki Urabe, Shuntaro Yoshimura, Motonari Ri, Koichi Yagi, Yasuyuki Seto

**Affiliations:** 1 Department of Gastrointestinal Surgery, Graduate School of Medicine, The University of Tokyo, Tokyo, Japan; 2 Gastrointestinal Surgery Division, Department of Surgery, Japanese Red Cross Omori Hospital, Tokyo, Japan; 3 Department of Gastroenterological Surgery, Mitsui Memorial Hospital, Tokyo, Japan; 4 Department of Gastroenterological Surgery, Gastroenterological Center, Cancer Institute Hospital, Japanese Foundation for Cancer Research, Tokyo, Japan; 5 Gastric Surgery Division, National Cancer Center Hospital, Tokyo, Japan

**Keywords:** lymphangiography, chylothorax, esophagectomy

## Abstract

**INTRODUCTION:**

Chylothorax following esophagectomy is a relatively rare but significant complication, with incidences ranging from 1.1% to 3.8%. It typically arises from damage to the thoracic duct or associated lymphatic vessels during extensive lymph node dissection and thoracic surgery. Initial management usually relies on conservative approaches such as dietary modifications, drainage, and pharmacotherapy. If these methods are ineffective, surgical intervention becomes necessary. However, standardized guidelines for the optimal management of thoracic duct injuries are as yet lacking, with decisions made on a case-by-case basis. We describe a case of chylothorax following esophagectomy where lymphangiography played a crucial role in guiding the surgical approach.

**CASE PRESENTATION:**

We report the clinical course of a 72-year-old male who developed chylothorax following esophagectomy for recurrent esophageal cancer. Despite initial conservative management, including octreotide and total parenteral nutrition, the patient’s condition required further intervention. Lymphangiography performed on postoperative day (POD)14 revealed a rare anatomical variation of the thoracic duct, leading to successful surgical ligation through a right cervical approach. The patient’s postoperative course was complicated by mediastinal fluid accumulation and resultant cardiac tamponade, necessitating emergency mediastinal drainage. Following the targeted surgical intervention, the drainage volume decreased, and the patient gradually resumed oral intake after swallowing function training. He was discharged on POD118.

**CONCLUSIONS:**

Thoracic duct injury after esophagectomy is challenging, especially with anatomical variations. Lymphangiography enables precise localization, guides surgery, and improves outcomes in chylothorax patients. The literature confirms that its early use reduces hospital stays and complications. Tumor invasion or inflammation at or beyond T3 may increase surgical complexity and injury risk. Given its diagnostic and therapeutic benefits, lymphangiography should be integrated into the standard protocols for chylothorax, especially in cases in where conservative treatment fails or anatomical variations are suspected.

## Abbreviations

CT
: computed tomography
ESD
: endoscopic submucosal dissection
MCT
: medium-chain triglyceride
NPO
: nil per os
POD
: postoperative day
TPN
: total parenteral nutrition

## INTRODUCTION

Chylothorax following esophagectomy is a relatively rare complication, with reported incidences ranging from 1.1% to 3.8%.^1–3)^ Chylothorax develops when the thoracic duct or associated lymphatic vessels are damaged, resulting in chyle leakage into the thoracic cavity. The risk of thoracic duct injury is especially high during esophageal cancer surgery due to extensive lymph node dissection and dissection around the thoracic duct, both of which are often required.

The initial management of chylothorax typically involves conservative approaches, such as fasting, a medium-chain triglyceride diet, drainage, and pharmacologic agents (e.g., octreotide). However, when conservative treatments are ineffective, surgical intervention is often necessary. Despite surgery being required in some cases, standardized guidelines regarding the optimal surgical techniques or timing for treating thoracic duct injuries have yet to be established. Management decisions are thus made on a case-by-case basis.

Herein, we describe a patient who developed chylothorax following esophagectomy. Lymphangiography was performed because thoracic duct injury was suspected intraoperatively, and this modality proved to be extremely useful in determining the optimal surgical approach. Lymphangiography facilitated identifying the course of the thoracic duct and the site of the injury, thereby guiding the selection of the most appropriate treatment strategy. We discuss the utility of lymphangiography in the management of chylothorax following esophagectomy in this report.

## CASE PRESENTATION

The patient was a 72-year-old male. He had undergone endoscopic submucosal dissection (ESD) for esophageal cancer 11 years prior and had since remained recurrence-free. During a follow-up esophagogastroduodenoscopy, a type 0-IIb lesion was identified 18 cm from the dental line. Computed tomography (CT) showed neither lymph node nor distant metastasis, and the diagnosis was cT1aN0M0 cStage 0 (JES 11th)/T1N0M0 Stage I (UICC 8th). The patient underwent another ESD.

Pathological examination revealed a type 0-IIb, 25 × 20 mm squamous cell carcinoma, classified as pT1a-MM, ly (0), v (1), pHM0, pVM0. Due to the presence of vascular invasion, additional treatment was recommended, and the patient opted for radical resection. He underwent a subtotal esophagectomy via a trans-hiatal and cervical approach, with three-field lymph node dissection, and reconstruction using a gastric conduit passed through the posterior mediastinum and anastomosed at the cervical region. During mediastinal manipulation, a thoracic duct injury was suspected, and a structure presumed to be the thoracic duct was therefore ligated.

**Fig. 1:**
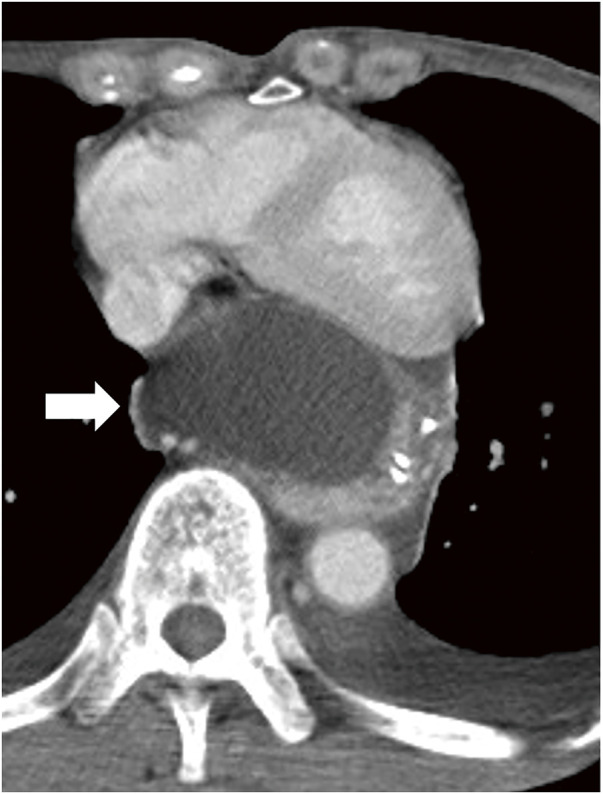
Mediastinal CT image on POD12. The low-attenuation area (white arrow) in the mediastinum, indicative of lymphatic leakage, compresses the pericardium.

On postoperative day (POD) 8, the patient developed a fever. By POD12, he exhibited tachycardia and hypotension. A CT scan revealed an increase in mediastinal fluid accumulation, raising suspicion of cardiac tamponade due to chyle leakage (**Fig. 1**).

**Fig. 2:**
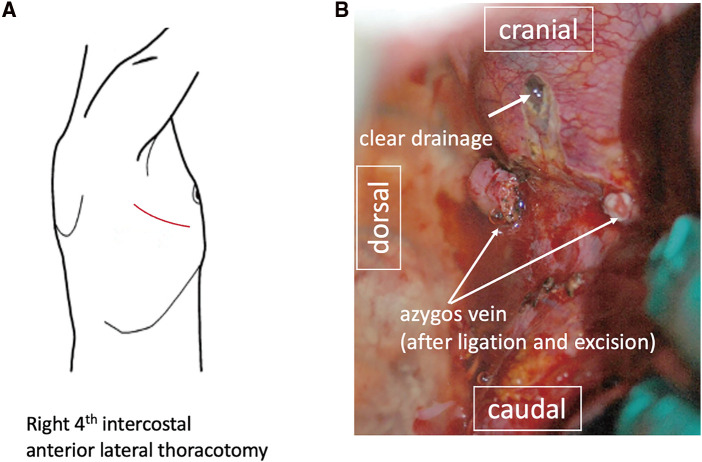
First reoperation performed on POD12 as an emergency procedure. (**A**) The patient was placed in the left lateral decubitus position, and a right-sided thoracotomy was made through the fourth intercostal space. (**B**) The tense upper mediastinum was intentionally perforated, revealing clear (lymphatic) fluid.

Emergency mediastinal drainage was planned. A right thoracotomy was performed through the fourth intercostal space. The mediastinum was accessed, and clear fluid was observed upon incision (**Fig. 2**). A drain was placed in the dissected mediastinum and right pleural cavity, the surgical procedures.

**Fig. 3:**
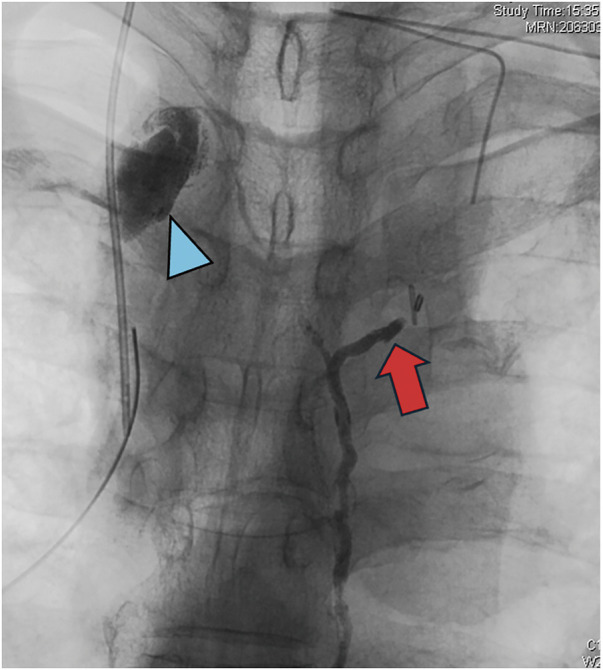
Upper mediastinal X-ray image during lymphangiography performed on POD14. The image shows the ligated left branch of the thoracic duct (arrow) from the initial surgery and contrast agent leakage from the stump of the right branch of the thoracic duct (arrowhead).

Lymphangiography was performed on POD14, as there was no marked change in drainage volume postoperatively. The lymphangiography revealed a rare anatomical variation, that is, the thoracic duct ascended on the right side of the descending aorta and bifurcated in the upper mediastinum (**Fig. 3**). Additionally, it was found that the ligation performed during the initial surgery had been of the left branch of the thoracic duct, while contrast leakage was observed from the right branch. To reduce the flow in the thoracic duct and promote occlusion of the leakage site, we administered octreotide, and nutritional management was provided with total parenteral nutrition (TPN). Despite ongoing management of the mediastinal drainage, there was little change in the drainage volume. Therefore, thoracic duct ligation was scheduled for POD41. Lymphangiography performed on POD14 revealed the thoracic duct injury to be located in the right upper mediastinum, leading to the decision to approach this site from the right cervical region.

**Fig. 4:**
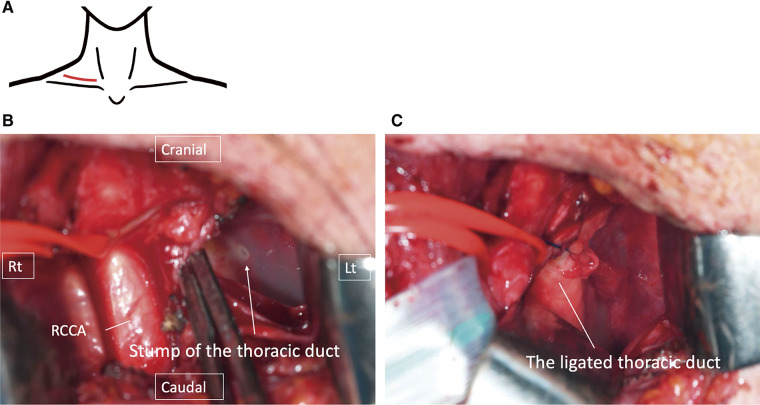
Second reoperation performed on POD41. (**A**) The surgery was conducted through a right cervical collar incision. (**B**) After retracting the right common carotid artery (RCCA) laterally, the stump of the right branch of the thoracic duct was identified. (**C**) The right branch of the thoracic duct was ligated.

**Fig. 5:**
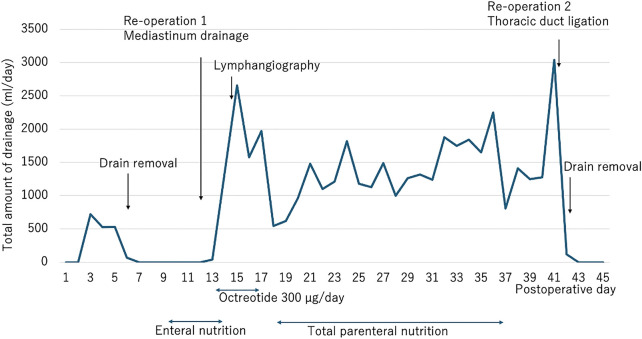
Relationship between the postoperative course of esophageal cancer and the amount of pleural effusion.

A collar incision was made along the superior border of the right clavicle. A cavity caused by lymph leakage was identified, between the trachea and the right recurrent laryngeal nerve, and was carefully preserved. Upon incision of the cavity, leakage of clear serous lymphatic fluid was observed. The site of thoracic duct rupture was identified within the cavity and ligated (**Fig. 4**). Following this procedure, the mediastinal and pleural drainage volumes both decreased significantly, leading to sequential removal of the two drains. The patient experienced decreased swallowing function, necessitating TPN management and swallowing function training until he was able to resume oral intake. He was discharged on POD118 (**Fig. 5**).

## DISCUSSION

### Thoracic duct injury

Thoracic duct injury following esophagectomy, though rare, presents a significant therapeutic challenge when it does develop. While the incidence is low, the complications associated with chylothorax, such as nutritional deficiencies and prolonged hospitalization, make this injury a critical clinical issue requiring prompt attention. The primary cause of thoracic duct injury is related to surgical manipulation within the thoracic and mediastinal regions. Even with meticulous attention to the typical anatomical course of the thoracic duct, injuries may still occur due to anatomical variability and the complexity of the procedures involved.

This risk is further exacerbated by the existence of individual anatomical variations, which can make the thoracic duct more susceptible to injury even when the surgeon remains mindful of its usual pathway.

When thoracic duct injury occurs, clinical management may present particularly challenging issues. Initial conservative measures, including dietary adjustments, drainage, and pharmacological agents, might be insufficient. In such cases, surgical intervention is generally necessary. However, determining the optimal timing and approach for surgery raises complex challenges, and widely accepted guidelines for such patients have yet to be established.

### Thoracic duct variations

**Fig. 6:**
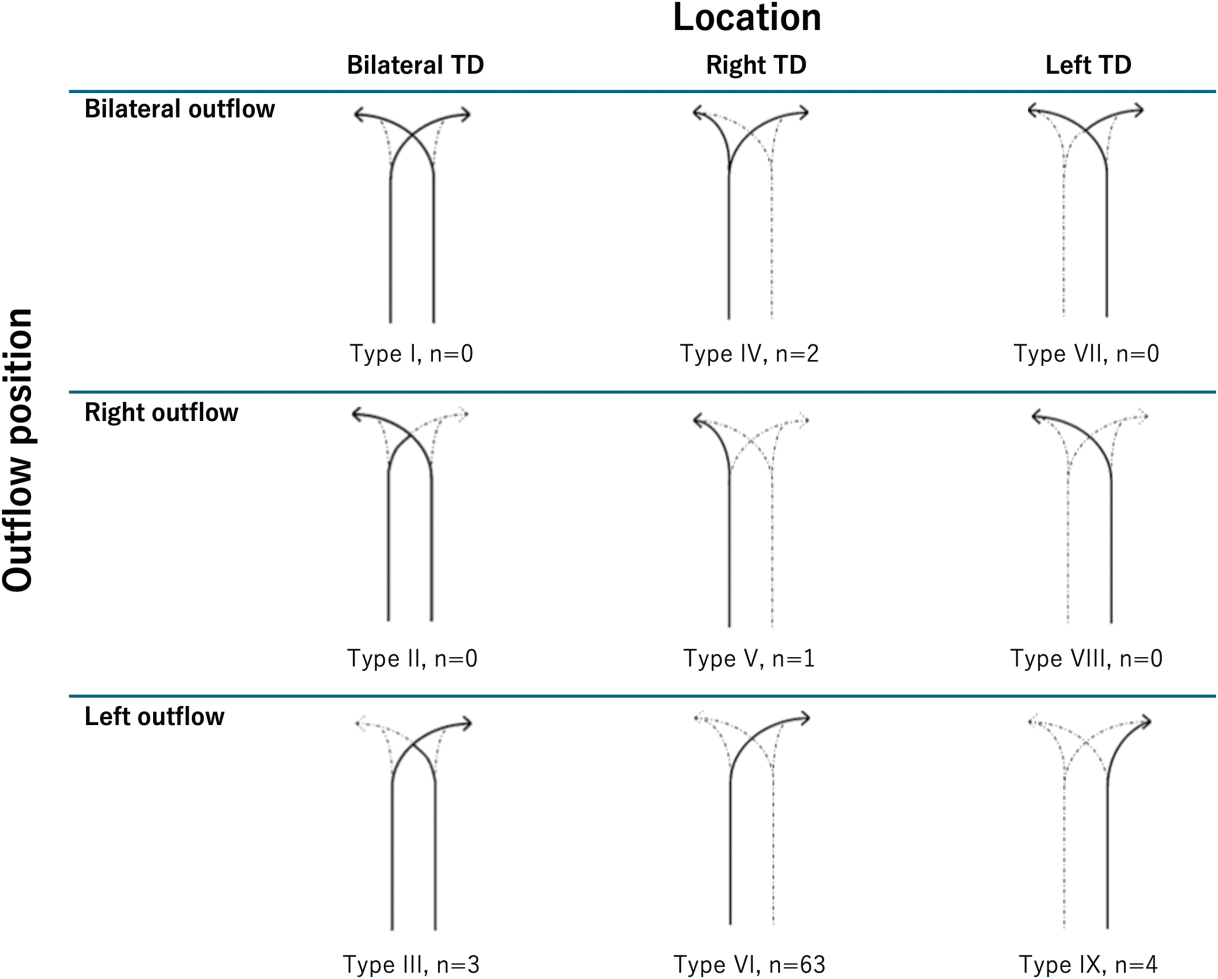
Classification of thoracic duct pathways according to Adachi and Kihara (n = 73). The most common pathway is Type VI, where the right thoracic duct drains into the left venous angle. In this case, the thoracic duct exhibited a rare Type IV pattern, where the right thoracic duct bifurcates into two branches (adapted and modified from Okuda et al.,^5)^ originally sourced from Adachi and Kihara,^4)^ reproduced with permission from Springer Nature).

Adachi et al. classified thoracic duct pathways into nine types (Type I–IX) based on their courses and sites of termination (**Fig. 6**).^4,5)^ By far the most common type, accounting for 86% of cases, is Type VI, wherein the right thoracic duct runs along the right side of the aorta and terminates at the left venous angle. In cases such as our present patient, the thoracic duct follows the rare Type IV pattern, which is seen in only 2.7% of cases. With this pattern, the duct bifurcates in the upper mediastinum.

### Lymphangiography

Lymphangiography can be performed via several different approaches, including direct access through lymphatic vessels in the dorsal foot, transabdominal access to the cisterna chyli, or retrograde access via the subclavian vein. In our present case, the inguinal lymph node was visualized employing ultrasonography and then punctured percutaneously, allowing injection of the oily contrast agent Lipiodol into the lymphatic vessels. Lipiodol has embolic properties, and there are case reports describing amelioration of chyle leakage after lymphangiography.

A PubMed search using the keywords “esophagectomy” and “lymphangiography” identified 25 case reports with chylothorax that underwent lymphangiography.^6–28)^ Of these, 23 described the postoperative course of esophagectomy with lymphangiography. Thus, along with the present case, a total of 24 cases were analyzed. In 21 of the 24 cases, the site of leakage was accurately detected, and 7 cases were found to have anatomical abnormalities. In 8 cases, the chyle leakage resolved with conservative treatment following lymphangiography. These findings raise the possibility that thoracic duct injury following esophagectomy tends to result in prolonged hospitalization, with a median duration of 35.5 days. However, following lymphangiography, the median time to drain removal was only 9 days. This suggests that early lymphangiography, when feasible, may facilitate establishing a treatment strategy and potentially reducing the hospital stay in cases with chylothorax.

It is noteworthy that complications related to lymphangiography are rare. Therefore, when unexpected chyle leakage occurs intraoperatively, anatomical abnormalities warrant consideration, and lymphangiography should be performed as this modality may serve as a therapeutic intervention providing diagnostic information (**Table 1**).

**Table 1:** Characteristics and outcomes of 24 cases

Characteristics		
Age	Median 65 (48–83)	n = 24
Unknown	0	
Gender		
Male	20	
Female	3	
Unknown	1	
Location of esophageal cancer		
Cervical	2	
Upper	1	
Middle	4	
Lower	10	
EGJ	1	
Unknown	6	
TNM classification (UICC 8th)		
T		
T1	1	
T2	2	
T3	7	
T4a	2	
T4b	2	
N		
N0	6	
N1	4	
N2	4	
N3	0	
M		
M0	14	
M1	0	
Unknown	10	
Stage (UICC 8th)		
I	1	
II	6	
III	4	
IVA	3	
IVB	0	
Unknown	10	
Surgical method		
Thoracotomy	10	
Thoracoscopy	7	
Robot (thoracic approach)	1	
Mediastinoscopy	1	
Unknown	5	
Preoperative treatment		
Chemotherapy	9	
CRT	3	
ESD	1	
Unknown	10	
Management of the thoracic duct at 1st ope		
Ligation	13	
Unknown	11	
Recognition of thoracic duct injury	Median 5.5 (0–23)	n = 20
Unknown	4	
Findings at first		
Drain	17	
Symptoms	6	
Unknown	1	
Lymphangiography performed		
Success	22	
Failed	2	
Unknown	0	
Contrast agent		
Lipiodol	19	
Ethiodol	2	
99 mTc HAS	1	
Unknown	2	
Days after surgery to lymphangiography	Median 13.5 (6–43)	n = 18
Unknown	6	
Puncture site for lymphangiography		
Inguinal	13	
Foot	5	
Para-aortic lymph node	1	
Transcervical	1	
Unknown	4	
Thoracic duct anomaly		
Rt outflow	1	
Bilateral outflow	1	
Duplicated	5	
None	6	
Unknown	11	
Treatment before lymphangiography		
TPN	14	
Octreotide	15	
Ethilefrine	1	
Try ligation	3	
Drainage	3	
Pleural adhesions	1	
Unknown	5	
Treatment after lymphangiography		
Conservative treatment	8	
Pleural adhesions	1	
Embolization	3	
Radiation	1	
Operation		
Thoracotomy	6	
Laparotomy	3	
Transcervical	1	
Unknown	5	
Times of re-ope after esophagectomy		
0 times	10	
1 time	8	
2 times	5	
Unknown	1	
Days from initial lymphangiography to drain removal	Median 9 (2–60)	n = 8
Unknown	16	
Site of leakage		
Cervical	2	
Superior mediastinal	3	
Inferior mediastinal	14	
Abdominal	2	
Not detected	3	
Unknown	0	
Complications after lymphangiography		
Abdominal discomfort (Clavien-Dindo Grade 1)	1	
Unknown	23	
Discharge day	Median 35.5 (7–124)	n = 20
Unknown	4	

Summary of the characteristics and outcomes of 24 cases, including 23 case reports identified through a PubMed search using the keywords “esophagectomy” and “lymphangiography,” as well as the present case.

Additionally, there was a correlation between the location of the esophageal cancer and the site of thoracic duct leakage. Of the 24 case reports analyzed, 14 included information on both tumor location and the site of Lipiodol leakage. When the boundary between the upper and lower mediastinum was defined as being at the level of the aortic arch, the tumor location and the site of leakage corresponded in all but 1 of these 14 cases. Most of the tumors were in fact located at or beyond the T3 level, where it is possible for tissue layer structures to be disrupted by tumor invasion and inflammation, potentially increasing the complexity of the surgical procedure and thereby increasing the risk of injury. Additionally, in two cases in which the preoperative diagnosis was a tumor at the T2 level, preoperative chemotherapy might have resulted in shrinkage of the tumor and surrounding tissues, including the lymphatic vessels, possibly associated with an unexpected course of the thoracic duct, although this may not represent a true anatomical variation.

### Surgical approach

When the patient shows little or no response to conservative management strategies, surgical ligation of the thoracic duct is often deemed to be necessary. Due to the anatomical characteristics of the thoracic duct, the approach, whether cervical, thoracic, or abdominal, must be carefully selected. Thoracotomy or video-assisted thoracoscopic surgery is widely used in making such choices.

Identifying the site of thoracic duct rupture and confirmation of the duct’s course using lymphangiography proved to be very useful in our present patient. The initial surgery involved reconstruction with a gastric tube passed through the posterior mediastinum, making an abdominal approach difficult. Lymphangiography revealed the site of leakage to be the right branch of the thoracic duct, at the level of the clavicle. Consequently, the decision was made to ligate the thoracic duct through a right cervical approach.

### Treatment flowchart for chylothorax

**Fig. 7:**
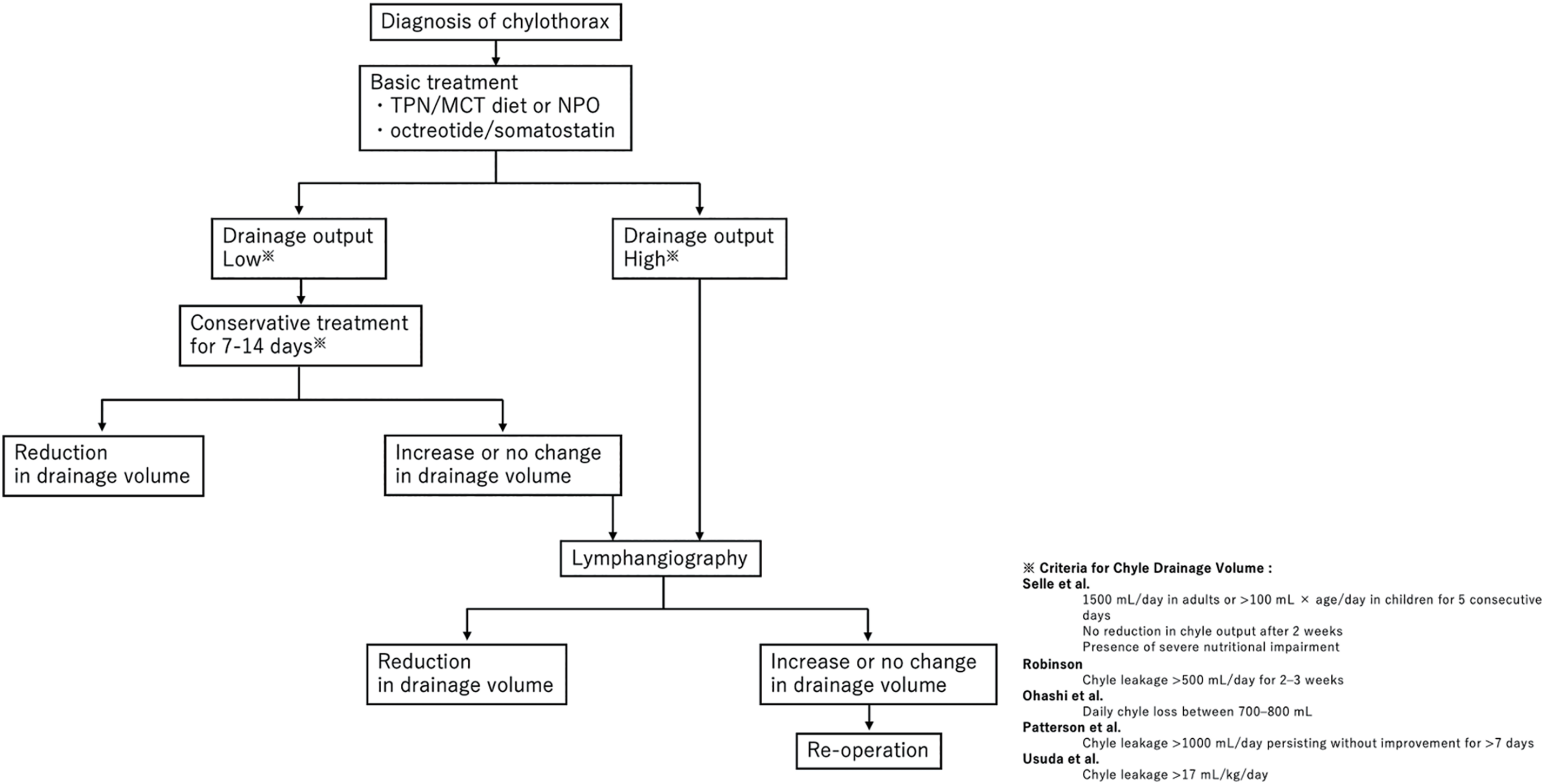
A flowchart for decision-making in the management of chylothorax is presented. In this case, the first reoperation was an emergency surgery performed for life-saving purposes, and catheter-related infection treatment was conducted after lymphangiography, which slightly deviated from the flowchart.^29–34)^ However, the overall course aligns with the flowchart, as lymphangiography was performed due to a high drainage volume, and ligation was carried out during the second reoperation because there was no change in the drainage volume.

**Figure 7** illustrates the treatment strategy for chylothorax following diagnosis. The fundamental approach includes TPN or a medium-chain triglyceride (MCT) diet or nil per os (NPO), in addition to octreotide or somatostatin administration.^29)^ The treatment course is determined based on the volume of chyle output. If the volume of chylothorax is low, conservative treatment should be administered for approximately 7–14 days. In cases resistant to conservative management or when the chyle output is high, early consideration of lymphangiography is warranted. Lymphangiography has a therapeutic embolization effect and may lead to resolution of chylothorax; however, if no improvement is observed, surgical ligation should be considered.

The classification of chyle output based on surgical indications proposed in previous studies, not limited to esophageal cancer, is as follows:

Selle et al.: Chyle output exceeding 1500 mL/day in adults or 100 mL × age/day in children for 5 consecutive days, persistent output for over 2 weeks, or severe nutritional impairment.^30)^

Robinson: Chyle leakage >500 mL/day for 2–3 weeks.^31)^

Ohashi et al.: Daily chyle loss between 700 and 800 mL.^32)^

Patterson et al.: Chyle leakage >1000 mL/day persisting without improvement for more than 7 days.^33)^

Usuda et al.: Chyle leakage >17 mL/kg/day.^34)^

In addition, out of the 24 cases (**Table 1**) we referred to, including our own, the number of days from the onset of chylothorax to lymphangiography was recorded in 18 cases. The median was approximately 6 days. Based on this, performing lymphangiography, rather than surgery, may be a viable option when chyle loss of around 1000 mL/day persists for approximately 1 week.

Ideally, as shown in this flowchart, lymphangiography should be performed to identify the site of leakage, and if the chyle leak does not improve afterward, surgical intervention should be considered immediately. However, in this case, management was complicated by catheter-related infections and anastomotic leakage, ultimately resulting in a delay of approximately 1 month before the second reoperation.

## CONCLUSIONS

Thoracic duct injury, though rare, is a challenging complication following esophagectomy. Significant morbidity may develop if duct injuries are not promptly and effectively managed. The variability in thoracic duct anatomy, as demonstrated in our present case, highlights the importance of considering lymphangiography when injury is suspected. Lymphangiography proved to be invaluable in this case, providing precise localization of the injury and the identification of a rare anatomical variant. This allowed us to select a targeted surgical approach, ultimately leading to successful resolution of the chylothorax.

The findings from this case and the literature review suggest that early lymphangiography should be considered in cases of suspected thoracic duct injury following esophagectomy, particularly when conservative management fails. This approach not only aids in diagnosis but can also guide the surgical strategy, potentially reducing the duration of hospitalization and improving patient outcomes.

In summary, lymphangiography plays a critical role in the management of thoracic duct injuries, providing both essential diagnostic information and therapeutic guidance. Given its safety and efficacy, lymphangiography should be integrated into the standard management protocol for chylothorax following esophagectomy, especially in complex or refractory cases.

## DECLARATIONS

### Funding

Not applicable.

### Authors’ contributions

YS was the lead surgeon for all three surgical procedures and supervised the overall management of the case.

KY, MR, SYo, and MU managed the patient postoperatively and contributed to the clinical decision-making throughout the treatment course.

MR assisted in all three surgeries, while MU assisted in the first surgery.

SYa provided critical guidance and revisions to the manuscript, ensuring its accuracy and intellectual rigor.

All authors have read and approved the final manuscript.

Additionally, all authors agree to be accountable for all aspects of the work, ensuring that questions related to the accuracy or integrity of any part of the work are appropriately investigated and resolved.

### Availability of data and materials

Not applicable.

### Ethics approval and consent to participate

Ethics approval: Not applicable.

Written informed consent for participation in this study was obtained from the patient.

### Consent for publication

Written informed consent was obtained from the patient for publication of this case report.

### Competing interests

The authors declare that they have no competing interests.
